# Microbial community roles and chemical mechanisms in the parasitic development of *Orobanche cumana*

**DOI:** 10.1002/imt2.31

**Published:** 2022-06-13

**Authors:** Jiao Xi, Beilei Lei, Yong‐Xin Liu, Zanbo Ding, Jiaxi Liu, Tengqi Xu, Lijun Hou, Siqi Han, Xun Qian, Yongqing Ma, Quanhong Xue, Jinming Gao, Jie Gu, James M. Tiedje, Yanbing Lin

**Affiliations:** ^1^ College of Life Sciences Northwest A&F University Yangling Shaanxi China; ^2^ State Key Laboratory of Crop Stress Biology for Arid Areas, Center of Bioinformatics Northwest A&F University Yangling Shaanxi China; ^3^ Institute of Genetics and Developmental Biology Chinese Academy of Sciences Beijing China; ^4^ Department of Natural Resource Sciences McGill University Montreal Quebec Canada; ^5^ Interdisciplinary Research Center for Soil Microbial Ecology and Land Sustainable Productivity in Dry Areas Northwest A&F University Yangling Shaanxi China; ^6^ State Key Laboratory of Soil Erosion and Dry Land Farming Institute of Soil and Water Conservation, Chinese Academy of Sciences and Ministry of Water Resources Yangling Shaanxi China; ^7^ College of Natural Resources and Environment Northwest A&F University Yangling Shaanxi China; ^8^ Shaanxi Key Laboratory of Natural Products & Chemical Biology Northwest A&F University Yangling Shaanxi China; ^9^ Center for Microbial Ecology Michigan State University East Lansing Michigan USA

**Keywords:** Cyclo(Pro‐Val), *Orobanche cumana*, parasitism plant, rhizosphere microbiota, sunflower

## Abstract

*Orobanche cumana* Wallr. is a holoparasite weed that extracts water and nutrients from its host the sunflower, thereby causing yield reductions and quality losses. However, the number of *O. cumana* parasites in the same farmland is distinctly different. The roots of some hosts have been heavily parasitized, while others have not been parasitized. What are the factors contributing to this phenomenon? Is it possible that sunflower interroot microorganisms are playing a regulatory role in this phenomenon? The role of the microbial community in this remains unclear. In this study, we investigated the rhizosphere soil microbiome for sunflowers with different degrees of *O. cumana* parasitism, that is, healthy, light infection, moderate infection, and severe infection on the sunflower roots. The microbial structures differed significantly according to the degree of parasitism, where Xanthomonadaceae was enriched in severe infections. Metagenomic analyses revealed that amino acid, carbohydrate, energy, and lipid metabolism were increased in the rhizosphere soils of severely infected sunflowers, which were attributed to the proliferation of *Lysobacter*. *Lysobacter antibioticus* (HX79) was isolated and its capacity to promote *O. cumana* seed germination and increase the germ tube length was confirmed by germination and pot experiments. Cyclo(Pro‐Val), an active metabolite of strain HX79, was identified and metabolomic and molecular docking approaches confirmed it was responsible for promoting *O. cumana* seed germination and growth. And we found that *Pseudomonas mandelii* HX1 inhibited the growth of *O. cumana* in the host rhizosphere soil. Our findings clarify the role of rhizosphere microbiota in regulating the parasite *O. cumana* to possibly facilitate the development of a new weed suppression strategy.

## Highlights

- Parasitic weed growth is influenced by microbes in the sunflower rhizosphere.
- The germination of *Orobanche cumana* seeds can be facilitated by *Lysobacter antibioticus* HX79 metabolites.
- The molecular docking strategy can be used for screening active chemicals.

## Graphical abstract

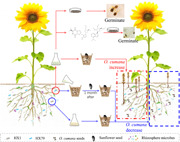

## INTRODUCTION

Within the angiosperm kingdom, parasitism has evolved numerous times, and parasitic weeds may now be found in a wide range of ecological environments worldwide [1, 2]. Parasitic plants can be obligatory or facultative parasites; they can adhere to the shoot or the root and can be hemi‐ or holoparasitic in nature. Parasitic plants take on several patterns to infect host plants. Some, like dodder (*Cuscuta* spp.) and mistletoe (*Arceuthobium* spp.), are aerial parts parasites, whereas Orobanchaceae, such *Orobanche* and *Phelipanche aegyptiaca*, reach the underground root and constitute one of the most damaging and significant threats to the agricultural economy. *Orobanche cumana*, an obligate root parasitic plant, causes great damage to economically important crops, such as Solanaceae, Compositae, and Cucurbitaceae plant families and affects total yields [3]. The life cycle of *O. cumana* has two stages, preparasitic and parasitic. The preparasitic stage comprises seed preconditioning followed by *O. cumana* seed germination, which is promoted by the presence of the chemical inducer strigolactones (SLs) released by host plant roots. However, the parasitic stage initiates with the parasite developing a special projection or root‐like structure known as haustorium that directly penetrates the tissues of a host and draws minerals and water and varying proportions of their carbohydrate requirements. After successful invasion and connection to the host root, the parasitic seedling grows into a bulbous structure known as a tubercle, from which a floral meristem emerges above the ground to produce flower and set seeds [4].

Because a wide diversity of microorganisms, including beneficial, harmful, and neutral microbes, are present in rhizosphere soils, they are likely to simultaneously interact with plant roots in the rhizosphere during plant growth and development [5]. Additionally, the occurrences of some diseases are associated with the stability of the microbial community [6], such as the microbial species, abundance, structural composition, and function [7, 8, 9]. Studies have shown that microbes can interfere in the life cycles of root parasitic weeds either by deterring the parasite or triggering processes that impair infection of the crop roots [10, 11]. Hemmati et al. [12] identified the pathogen *Talaromyces trachyspermus* as a promising biocontrol agent for *O. ramose* reduction. Kruh et al. [13, 14] isolated *Pseudomonas* strain *PhelS10* from tomato roots, which suppressed the germination of *P. aegyptiaca* seeds by 80%. According to Chen et al. [15], *Streptomyces enissocaesilis* culture filtrate drastically lowered the germination rate of *O. cumana* by 40%. Above all, these approaches imply that *O. cumana* parasitism can be suppressed by microorganisms. They do not, however, explain how microorganisms inhibit *O. cumana* parasitism, and the underlying parasite processes are not adequately defined.

Host‐derived chemical signals, specifically SLs, trigger seed germination, which is perceived by weeds via SLs receptors and serves as a signaling molecule for the rhizosphere microbes [16, 17]. Many studies have aimed to screen and identify synthetic compounds targeted at SLs receptors to reduce weed parasitism by suicide germination [18, 19, 20], such as sphynolactone‐7, a specific femtomolar‐range suicide germination stimulant for *Striga hermonthica* [21], and β‐lactones, potent irreversible antagonists of SLs receptors [22]. It is considered that SLs trigger *O. cumana* seed germination through seven members of an independently diverged group of α/β hydrolase‐fold receptors called KARRIKIN‐INSENSITIVE2 (*KAI2*, referred to as “*KAI2*s” in the following) [23]. The divergence of the ligand pockets formed in *OcKAI2*s during their evolution is beneficial for allowing seeds to detect the structurally variable SLs exuded by their preferred host species. We hypothesize a microbe‐mediated mechanism that involves the induction or inhibition of seed germination via microbial secondary metabolites without the presence of a host. Exploring the active metabolites act as germination inducers of parasitic weeds from screened effective microbial species is helpful for understanding the modes of interaction between parasitic seeds and bacteria. Untargeted metabolomic analysis to identify metabolites combined with molecular docking can be used to predict the potential interactions between metabolites and *KAI2*s.

*O. cumana* is a particular parasite of sunflower; however, its prevalence varies dramatically over the same farming region in Northwest China, ranging from parasitism‐free to severely infected. The same situation occurred in other weed‐infested farmlands, but it is unclear why this is the case, whether a specific microbe is associated with this phenomenon, and the possible mechanism underlying such association. Thus, the goal of this study was to discover the relationships between *O. cumana*'s parasitic activity and the composition and distribution of the rhizosphere microbiome around the host sunflowers. We identified the major microbial species involved in *O. cumana* parasitism and gained insight into the molecular recognition mechanism at work. Our analysis process might be used to explore different parasitic weeds, perhaps assisting in the development of alternative and sustainable parasitic weed management tactics.

## RESULTS

### Changes in microbiota according to the number of parasites

We determined whether the bacterial community composition in the rhizosphere correlated with parasitism of the host sunflower by *O. cumana* based on samples of the rhizosphere microbiota in the host flowering stage at three farm locations in Bayannur City, Inner Mongolia Autonomous Region, China (Figure S1A), where the samples were analyzed using the 16S ribosomal RNA (rRNA) amplicon sequencing technique. Remarkably, the microbiota communities in the parasitized and nonparasitized soils differed significantly. Unconstrained principal coordinate analysis (PCoA) showed that the rhizosphere microbiota formed two distinct clusters in the presence or absence of *O. cumana* parasitism (Figure S1B). The parasitic rhizosphere microbiota community exhibited relatively less diversity than the nonparasitized and controlled rhizosphere microbiota according to α‐diversity analysis (Figure S1C). Furthermore, Verrucomicrobia, Actinobacteria, and Gemmatimonadetes were more abundant in the parasitized sunflower roots than the nonparasitized sunflower roots (Figure S1D, false discovery rate adjusted *p* < 0.05, Wilcoxon rank‐sum test; Table S4). These findings confirmed the close association between the microbiota and parasitism by *O. cumana*.

**Figure 1:**
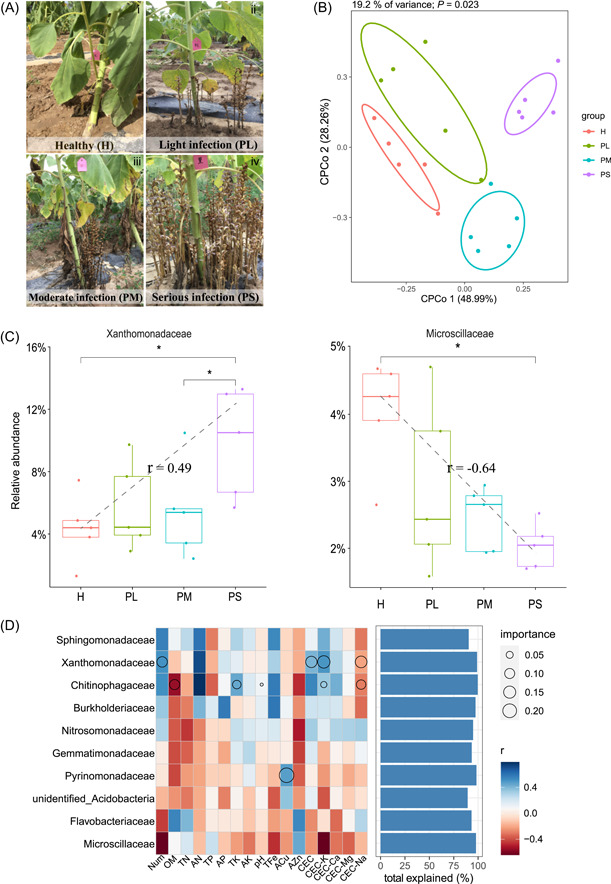
Differences in root microbiota with different levels of *Orobanche cumana* parasitism. (A) Four levels of *O. cumana* parasitism in sunflowers. Healthy (i), light (ii), moderate (iii), and severe (iv) parasitism corresponding to 0, 1–25, 26–50, and more than 50 parasites on sunflower roots, respectively. (B) Constrained principal coordinate analysis showing differences in microbial structures based on Bray–Curtis distances among healthy (H), light infection (PL), moderate infection (PM), and severe infection (PS) soils. (C) Abundances of Xanthomonadaceae and Microscillaceae (Wilcoxon test, *r*: Spearman correlation coefficient, *p* < 0.05). (D) Contributions of the number of *O. cumana* (Num) and soil properties to differences in the microbial community compositions based on correlations and the best multiple regression model. Circle sizes represent the importance of variables and colors represent Spearman's correlation coefficients. Abbreviations of soil properties: ACu, available copper; AK, available potassium; AN, available nitrogen; AP, available phosphorus; CEC, available zinc, cation exchange capacity; CEC‐Ca, cation exchange capacity for calcium; CEC‐K, cation exchange capacity for potassium; CEC‐Mg, cation exchange capacity for magnesium; CEC‐Na, cation exchange capacity for sodium; OM, organic matter; TFe, total iron; TK, total potassium; TN, total nitrogen; TP, total phosphorus.

After determining that the parasitism of sunflower by *O. cumana* was related to the host rhizosphere microbiota, the bacterial compositions were examined at different levels of parasitism to identify specific bacteria with possible associations. The compositions of the root bacterial microbiota in the light infection (PL), moderate infection (PM), and severe infection (PS) (parasitized) soils and H (healthy) host soils indicated relationships with the level of parasitism. Samples with the same level of parasitism were clearly clustered and separated from those with other levels of parasitism according to constrained PCoA (CPCoA) (Figure 1B, 19.2% of the explained variance, *p* = 0.023). In addition, Figure 1B shows that the samples tended to cluster together more closely as the level of parasitism increased, where the communities were more similar in the severely parasitized soil samples. Statistical analysis of the community composition for the 10 top‐ranked families demonstrated the greater abundances of Xanthomonadaceae (Wilcoxon test, *p < 0.05; Figure 1C), Burkholderiaceae, and Sphingomonadaceae but relatively lower abundances of Microscillaceae and Flavobacteriaceae (Figure S2 and Table S5) in the parasitized samples compared with the nonparasitized samples. Furthermore, 10 families differed significantly after comparing the microbiota in the PS and H soils (*p* < 0.05, absolute difference between means >0.3%), where Xanthomonadaceae was most abundant (Figure S3), thereby indicating that bacteria associated with the promotion of parasitic weed germination probably belonged to Xanthomonadaceae.

To determine whether the soil properties and the number of parasites had specific relationships with the composition of the microbial community, we calculated the correlations and the best multiple regression model between the relative abundances of microbial families and the differences in the soil properties for H, PL, PM, and PS for each pairwise set of soil samples. The number of parasites, available copper (ACu), cation exchange capacity (CEC), and cation exchange capacity for potassium (CEC‐K) were strong positive predictors of dissimilarities in the microbial communities and differences in the relative abundances of most families (Figure 1D). In particular, the number of *O. cumana* parasites was significantly correlated with the abundance of Xanthomonadaceae and the CEC and CEC‐K contents.

### Microbiome function analysis and prediction of biomarkers

**Figure 2:**
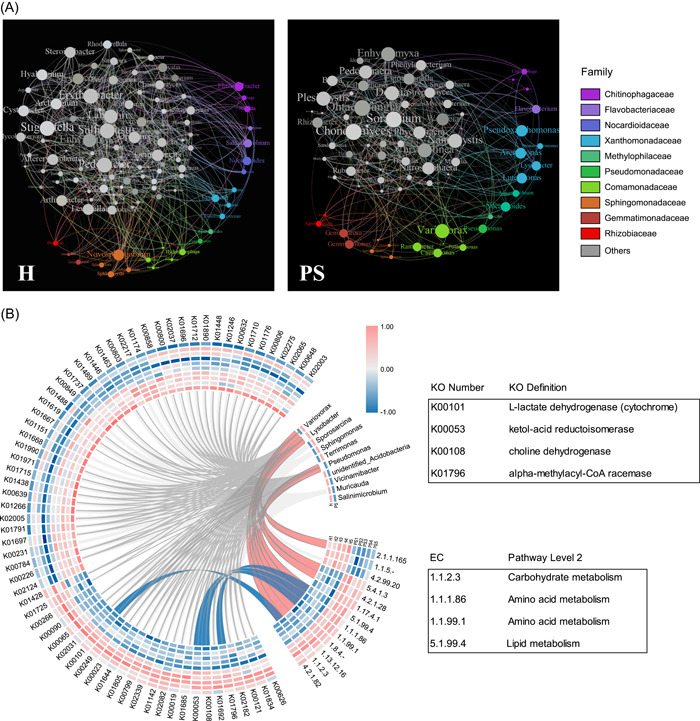
Co‐occurrence network and analysis of functional categories between H and PS. (A) Network showing co‐occurrence of genera (relative abundance >0.01%) under H and PS. The size of each node is proportional to the number of connections (degree). (B) Circos plot showing the relationships between ECs, KO functional orthologs, and top 10 bacteria. The functions represented by red lines correspond to the type of bacteria based on the KO functions. KO functional orthologs represented by blue lines correspond to KEGG enzymes. Gray lines represent KO functional orthologs corresponding to the top 10 bacteria. The heat map was drawn based on data obtained by MetaStats analysis (*q* < 0.05). The periphery of the heat map shows the PS group. The inner circle shows the H group. The outermost circle compares the relative abundances of the top 10 bacteria. EC, KEGG enzyme; H, healthy; KO, KEGG ortholog; PS, severe infection.

Comparisons were conducted at the taxa level between the H and PS samples to obtain further insights. The relationships among microbial taxa were estimated by constructing correlation networks for the genera with relative abundances greater than 0.1% based on metagenomic sequencing of H and PS (Figure 2A and Table S6). The network obtained for H contained 117 nodes and 627 edges with an average clustering coefficient of 0.583, whereas the network produced for PS only contained 82 nodes and 341 edges with an average clustering coefficient of 0.460. Thus, the network obtained for H appeared to have a more complex community structure, which may have contributed to greater adaptation and responses to environmental stresses. In addition, the greater abundance of Xanthomonadaceae in PS (3.37% in PS compared with 1.67% in H) may be associated with a higher level of parasitism.

Next, KEGG enzyme (EC) and KEGG orthology (KO) functional analyses were performed for the top 10 bacteria, as shown in the Circos plot in Figure 2B. A total of 13 ECs and 67 KOs (Table S7) differed significantly between H and PS. According to the heat map, 10 enzymes (Table S8) with higher abundances in PS (>80.0%) were mainly involved in metabolic pathways comprising amino acid metabolism (EC 1.1.1.86 and EC 1.1.99.1), carbohydrate metabolism (EC 1.1.2.3, EC 4.2.1.28, and EC 4.2.1.82), energy metabolism (EC 5.4.1.3 and EC 1.13.12.16), and lipid metabolism (EC 5.1.99.4). Research showed that valine, leucine, glycine, and threonine were affected by germination and radicle growth by *O. cumana* [24, 25]. The KOs and ECs were strongly correlated with bacteria comprising *Lysobacter*, *Variovorax*, and *Pseudomonas* (Figure S4). Therefore, we considered that these bacteria could be related to *O. cumana* germination and parasitism.

### Culturable bacteria related to *O. cumana* parasitism

**Figure 3:**
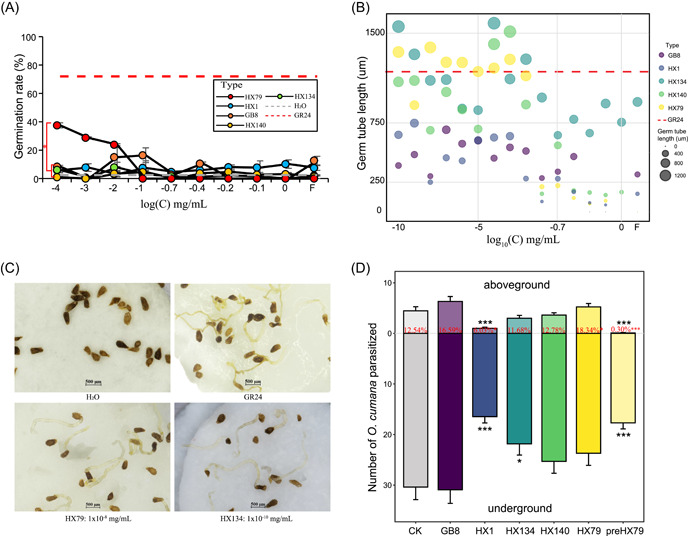
Effects of bacterial inoculation on germination and parasitism by *Orobanche cumana*. (A) Germination rate of *O. cumana* after adding metabolites from bacterial strains (HX1, HX79, HX134, HX140, and GB8), water (H_2_O, negative control), and GR24 (synthetic strigolactone analog as a positive control). The *X*‐axis represents the logarithm of the concentration. (B) Germ tube length. The red dashed line represents the average germ tube length with GR24. (C) Germination of *O. cumana* seeds after HX79 and HX134 were added. (D) Number of *O. cumana* parasitized. Percent with red represents the percentage aboveground number (%), that is, the ratio of the aboveground number relative to the number of all parasitic *O. cumana*. CK: *O. cumana* seeds but no bacteria added; NK: no seeds, no bacteria; GB8, HX1, HX134, HX140, and HX79: seeds and corresponding bacterial fermentation liquid added during the growth period; preHX79: seeds and fermentation liquid added 1 month ahead, but do not add during the growth period. Different letters denote significant difference among groups (*n* = 13, Kruskal–Wallis test, *p < 0.05, ***p < 0.001).

After isolation and screening used luria‐bertani (LB) medium, and analysis of the 16S rRNA gene sequences for bacteria in the sunflower rhizosphere, we obtained 70, 52, 63, and 97 bacterial strains from H, PL, PM, and PS, respectively. To determine the specific bacteria that promoted or inhibited parasitism by *O. cumana*, five strains comprising HX79 (*Lysobacter antibioticus* [Xanthomonadaceae] from PS soil), HX1 (*Pseudomonas mandelii* [Pseudomonadaceae] from H soils), HX134 (*Pseudomonas brassicacearum* subsp. *neoaurantiaca* from PL soil) and HX140 (*Pseudomonas chlororaphis* subsp. *chlororaphis* from PL soil), and GB8 (*Variovorax paradoxes* [Burkholderiaceae] from PS soil) were tested in seed germination assays and pot experiments. HX79 clearly affected the seed germination activity at concentrations of 1 × 10^–2^–1 × 10^–4^ mg/ml and the highest germination rate was about 40% (Figure 3A; the concentrations are shown in Table S9). The germ tube length in *O. cumana* has a crucial effect on the attachment of germinated seeds to the host roots [26], and thus successful parasitism. Statistical analysis of the germ tube lengths for the germinated seeds showed that they were longer with HX79, HX134, and HX140 than GB8 and HX1 (*p* < 0.05, Figure 3B,C, Table S9). Thus, HX79 promoted seed germination and enhanced the length of the germ tube in the seed germination process. The pot experiments showed that HX1 dramatically reduced the percentage aboveground number (%) of *O. cumana* that emerged, with 6.03% per host, whereas HX79 and GB8 increased the numbers of *O. cumana* parasitized plants (18.3% and 16.6% per plant, respectively; Figures 3D and S5). *O. Cumana* weeds suffer from suicide germination processes when HX79 fermentation liquid is added 1 month before sowing sunflower, and the growth of the parasite *O. Cumana* is ended early. As a result, the number of parasitic weeds is significantly reduced. The replicate experimental results were similar (Figure 3D).

### Cyclo(Pro‐Val) metabolite produced by HX79 stimulated *O. cumana* seed germination

**Figure 4:**
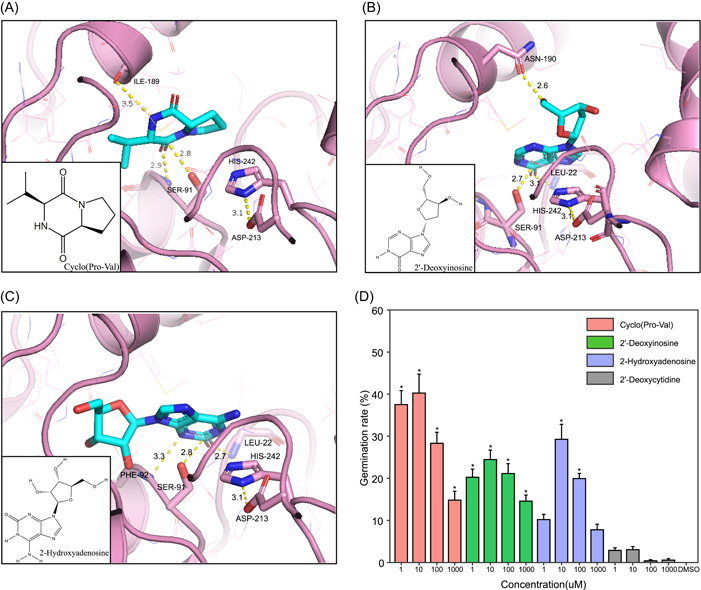
Predicted binding modes of three compounds and effects on *Orobanche cumana* seed germination rate. Chemical structures of Cyclo(Pro‐Val) (A), 2′‐deoxyinosine (B), and 2‐hydroxyadenosine (C) and visual representations of the ligand pockets in *O. cumana KAI2d2* proteins. Catalytic triad residues are shown as pink sticks. The metabolite is shown in cyan. All hydrogens are hidden. The distances between atoms involved in key interactions are shown by yellow dashed lines. (D) Germination rate of *O. cumana* after adding three test compounds (Cyclo(Pro‐Val), 2′‐deoxyinosine, and 2‐hydroxyadenosine) and one control compound (2′‐deoxycytidine) at concentrations of 1, 10, 100, and 1000 μM. Kruskal–Wallis test comparison with dimethyl sulfoxide, *p < 0.05.

About 500 compounds were detected in the HX79 culture solution by untargeted metabolomics analysis using semi‐quantitative analysis tests. Compounds with relative contents ranked in the top 50 (Table S10) were investigated to explore their possible interaction with the receptor *OcKAI2d2*. Finally, three compounds comprising Cyclo(Pro‐Val) (CAS: 5654‐87‐5), 2′‐deoxyinosine (CAS: 890‐38‐0), and 2‐hydroxyadenosine (CAS: 1818‐71‐9) were purchased from TargetMol, and their binding modes were predicted (Figure 4). Figure 4A shows that Cyclo(Pro‐Val) can bind in the active pocket of *OcKAI2d2* via two hydrogen bond interactions (carbonyl O on Cyclo(Pro‐Val) with the backbone NH on Ser 91 and NH on Cyclo(Pro‐Val) with the backbone carbonyl O on ILE 189). Ser 91 may nucleophilically attack the carbonyl C with a close distance of 2.8 Å to possibly induce a conformational transformation by the receptor to subsequently initiate the signaling pathway.

We also purchased the reference compound 2'‐deoxycytidine (CAS: 951‐77‐9) from the same company, which has no interaction with the *OcKAI2d2* receptor. The *O. cumana* seed germination activities were also tested. The assay results with Cyclo(Pro‐Val) showed that it significantly promoted the germination of *O. cumana* seeds with a germination rate of 40.26% at a concentration of 10 μM (Figure 4D). It should be noted that the germination rate with Cyclo(Pro‐Val) was equal to the overall germination rate with the HX79 cell‐free fermentation broth, thereby supporting our results. To confirm that the compound Cyclo(Pro‐Val) was produced by bacterial strain HX79, the detection was conducted using a Qtrap 5500 triple quadrupole system (AB SCIEX) and 5.43 mmol of metabolite was obtained per liter of broth (Figure S6). Thus, the results showed that bacterial strain HX79 metabolite Cyclo(Pro‐Val) could promote *O. cumana* seed germination and it may explain the biological activity of HX79.

## DISCUSSION

In this study, we examined the mechanism of microbial regulation of parasitic plant growth using *O. cumana* as an example. The soil rhizosphere microbiota in sunflower roots parasitized and non‐parasitized by parasites from three distinct farmlands were studied. The results showed that the rhizosphere microbiota differed between the parasitized and nonparasitized soil samples (Figure S1). At varying degrees of parasitism, specialized biomarker taxa such as Xanthomonadaceae were identified (Figure 1). Furthermore, we used metagenomic sequencing to assess the metabolic functions of the microbiota and discovered that the root microbiota in PS had greater metabolic enzyme activity and metabolic pathways than H. Previous studies have shown that *O. cumana* development and growth require large amounts of organic chemicals and energy, which are provided by the rhizosphere microbiota [27, 28]. The strength of species interactions determines biodiversity and the stability of microbial communities [29]. More interactive connections were found in the microbial communities in the H soils. *Lysobacter* species production of amino acid‐derived antibiotics has potent antibacterial or antifungal activity. This is explained by the low number of species nodes and edges in the soil of the PS group in the network analysis. If environmental perturbations occur, such as changes in the species present or pathogen invasion, microorganisms on healthy hosts can possibly suppress negative effects [30]. Cyclo(Pro‐Val) was constructed exclusively with highly repeated units of hydrophobic l‐amino acid residues, which showed moderate antifungal [31]. This explains that the microbial community in the PS group exhibits more abundant amino acid and energy metabolism pathways due to the abundance of the metabolite Cyclo(Pro‐Val) secreted by HX79. Based on the EC and KO functional orthologs correlated with bacteria, we identified five strains for testing in seed germination experiments. HX1 fermentation solution significantly reduced weed parasitism in pot tests. However, the mechanism was difficult to decipher due to the chemical mechanism that inhibits germination has not been clearly studied. On the contrary, the seed germination and pot studies revealed that the HX79 increased *O. cumana* germination and germ tube expansion (Figure 3). In summary, we have convincingly described why certain sunflower roots parasitize *O. cumana* while others do not in the same location using microbial ecology. Changing the bacterial community composition in the host rhizosphere soil may help to control *O. cumana* parasitism, and this process may be suitable for controlling other parasitic weeds.

The proximity of the germ tube and host roots is important for successful tubercle and development, and previous studies have demonstrated the chemotropic response of broomrape radicles to host root exudates [26, 32]. By establishing *KAI2d2* as the binding mode, it was possible to screen compounds for their ability to stimulate seed germination, and compounds Cyclo(Pro‐Val), 2′‐deoxyinosine and 2‐hydroxyadenosine were found to be effective. Moreover, Cyclo(Pro‐Val) exhibited the strongest germination effect. SLs represent a class of compounds with a butenolide ring (D ring) connected to an enol–ether that is conjugated with a carbonyl group, which was proposed to contribute to key structural requirements for seed germination activity. Although Cyclo(Pro‐Val) does not contain any fragment of the structure in SLs, it was proposed that the carbonyl of Cyclo(Pro‐Val) may react with Ser 91 by the nucleophilic attack and mimic the SLs to generate the seed germination activity. This finding revealed that not only SLs can stimulate *O. cumana* germination but also the metabolites of microorganisms can influence the germination of *O. cumana*.

We analyzed the chemical compositions of the soil samples and determined the correlations between environmental factors and the number of *O. cumana*. The results showed that the soil potassium concentration was higher when the number of infected plants was greater and the phosphorus concentration was lower. These changes in the availability of nutrients may be important for microbial community structure and *O. cumana* parasitism. Potassium [33], nitrogen, and phosphorus [34] depletion were previously shown to be directly responsible for damage to the host by *Orobanche*. Thus, changes in the soil nutrient contents may also affect the structure of the microbiome. Modification of the microbial community composition by adjusting the physicochemical properties of the soil, hence controlling *Orobanche* parasitism. This is a new perspective on the control of parasitic weeds.

## CONCLUSION

To investigate the mechanism controlling the parasitism of *O. cumana*, we performed an integrated multiomics analysis in this study. It was demonstrated that rhizosphere microbes have a regulatory effect on the parasitism of *O. cumana*. By constructing a molecular binding model, we successfully predicted three compounds that promote the germination of *O. cumana* and clarified their molecular recognition mechanisms. That is, microbe's metabolites include molecules that may recognize the receptor protein *OcKAI2*s of *O. cumana*, thereby inducing the germination of parasitic weeds. For practical purposes, the strategy of screen compounds appears applicable to other parasitic weeds, including *Phelipanche* species.

## METHODS

### Site description and sampling procedure

Thirteen groups of sunflower rhizosphere soil and root zone soil samples were collected from agricultural land, which had been previously cultivated with sunflower for more than 15 years. The sampling sites ranged from 40.15°N to 40.41°N and 106.57°E to 106.58°E at 1001 m above sea level, and they were located in Bayannur City, Inner Mongolia Autonomous Region, China (Table S1), which is a typical sunflower growing region with high yields in China. During July to August 2018 (the flowering stage), the samples were collected from three farmlands designated as A, B, and C, each with control, no parasitic, and parasitic three types. Nine groups of soil samples were collected, each with three replicates (Figure S1A). And then, the rhizosphere soils were second collected with five replicates and assigned to four grades comprising H PL, PM, and PS, which corresponded to 0, 1–25, 26–50, and over 50 *O. cumana* parasites, respectively, on one sunflower in farmland B (Figure 1A). The rhizosphere soil and root zone soil were obtained at a depth of 0–15 cm. Each soil sample was divided into three parts. The root zone soil samples were air‐dried, ball‐milled, and homogenized, before analyzing the physicochemical characteristics. The rhizosphere soil samples were divided into two parts, where one was stored at 4°C for bacterial isolation, screening, and identification, and the other was stored at –80°C for DNA extraction.

### Isolation and identification of culturable microorganisms

The sunflower rhizosphere soil was serially diluted and soil suspensions with different concentrations were evenly coated on the medium. LB medium was used to isolate bacteria at soil suspension concentrations of 10^–3^ and 10^–4^ in triplicate. After cultivation for 3–7 days, the medium was transferred to Petri dishes for counting, isolation, purification, and identification. Single colony‐forming units were selected and amplified based on the 16S rRNA gene with the bacterial universal primers 27F/1492R [35]. After detection by agarose gel electrophoresis, the reaction products were purified and sequenced with a Veriti 96‐Well SimpliAmpTM Thermal Cycler (Applied Biosystems). The 16S rRNA gene sequences were aligned using NCBI (https://www.ncbi.nlm.nih.gov/) Nucleotide BLAST to determine the approximate phylogenetic affiliations of the strains.

### Physicochemical characteristics of soil samples

The physicochemical characteristics of the root zone soil samples were determined to evaluate the soil conditions in the study field. Standard methods were used to measure the soil pH, total nitrogen, total phosphorus, total potassium, organic matter, available nitrogen, available phosphorus, available potassium, total iron, ACu, available zinc, CEC, CEC‐K, CEC for sodium, CEC for calcium, and CEC for magnesium as described in previous studies [24] (Table S2).

### DNA extraction and sequencing

Total genomic DNA was extracted from the soil samples using a FastDNA SPIN Kit for Soil (MP Biochemicals) according to the manufacturer's protocol. The prepared DNA samples were submitted to Novogene Co., Ltd. for library preparation and shotgun sequencing. Polymerase chain reaction assays were performed with the 515F (5′‐GTGCCAGCMGCCGCGGTAA‐3′) and 907R (5′‐CCGTCAATTCCTTTGAGTTT‐3′) primer pair [36], which amplified variable regions 4 and 5 in the 16S rRNA gene, and 16S rRNA amplicon sequencing was performed with the IonS5^TM^ XL platform (Thermo Fisher Scientific). The raw single‐end reads were processed in the following steps using USEARCH: relabeling sequencing names, removing barcodes and primers, filtering low‐quality reads, and identifying nonredundant reads [37, 38]. Unique reads were clustered into operational taxonomic units (OTUs) with ≥97% similarity. Representative sequences were selected with UPARSE [39] and aligned to the SILVA database (release 132) [40] using the Mothur algorithm [41] to annotate taxonomic information. To study the phylogenetic relationships among different OTUs and the different dominant species in each of the samples (groups), multiple sequence alignment was conducted using MUSCLE software (version 3.8.31) [42]. Metagenomic sequencing was performed with the Illumina PE150 platform (Illumina Inc.) by Novogene Co., Ltd. The Clean Data is assembled and analyzed by MEGAHIT software (v1.0.4‐beta). Then interrupted the assembled Scaftigs from N connection and left the Scaftigs without N [43, 44, 45]. All samples' Clean Data were compared to each scaffold, respectively by Bowtie2.2.4 software to acquire the PE reads not used [43]. All the reads not used in the forward step of all samples were combined and then used the software of SOAPdenovo (V2.04)/MEGAHIT (v1.0.4‐beta) for mixed. Break the mixed assembled scaffolds from the N connection to obtain the scaffolds. Filter the fragment shorter than 500 bp in all of Scaftigs for statistical analysis. The analysis pipeline for the metagenomic data was as described in previous studies [37, 38].

### *O. cumana* **seed germination**

*O. cumana* seeds were surface sterilized with 1% sodium hypochlorite solution [46]. *Lysobacter antibioticus* HX79 and *Variovorax paradoxus* GB8 were screened from PS soil samples, *Pseudomonas mandelii* HX1 from H soils, and *Pseudomonas brassicacearum* subsp. *neoaurantiaca* HX134 and *P. chlororaphis* subsp*. chlororaphis* HX140 from PL soil samples (Table S3). These strains were used in seed germination tests. The cell‐free fermentation broth was obtained by high‐speed centrifugation and suction filtration after culturing in an LB medium for 7 days. The methods used for pre‐conditioning *O. cumana* seeds and determining the cell‐free fermentation effects of *O. cumana* seeds were as described by Chen [15] and Ye [47].

### Pot experiments

Pot experiments were conducted in the Mobile Water Control Crop Shed, Research Institute of Arid Area Water‐saving Agriculture, Northwest A&F University. The soil comprised arable soil from farmland in Yangling, Shaanxi. *O. cumana* seeds were collected from a parasitized sunflower farm in Inner Mongolia. The pots were almost cylindrical with a diameter of 24.5 cm and a height of 27.5 cm. Each pot contained 10 kg of mixed soil. One kilogram of mixed soil contained 3.4 mg *O. cumana* seeds, 5% organic fertilizer, 0.43 g urea, and 0.15 g single superphosphate. In this experiment, there are two controls, one is added seeds, and the other is added water. At the same time, treatment groups and two controls were cultivated with the sunflower plants, with 13 replicate pots. Each pot was planted with three sunflower seeds and one was retained from the seedling stage. Weeding and irrigation were performed regularly to ensure normal plant growth. Every 2 weeks, irrigation was performed with 200 ml of 100‐fold diluted bacterial fermentation liquid. The sunflower plants were harvested in the grain filling stage (after growth for about 70–80 days). The height and dry weight of the host as well as the aboveground number, underground number, and dry weight of *O. cumana* were recorded. The soil was returned to the pot and the experiment was repeated 60 days later [15, 32].

### Extraction of metabolites from bacterial strain HX79, identification, and verification as active material

Bacterial strain HX79 was cultured in LB medium for 7 days in three replicate bottles. Next, 50 ml of the fermentation broth from each bottle was placed in a sterile sealed plastic bottle. The bottles were transported under low‐temperature preservation conditions to Metware Biotechnology Co., Ltd. for metabolite extraction and analysis. The sample extraction procedure involved thawing the sample and vortex mixing for 10 s. Next, 0.3 ml of the sample was placed in a 2 ml centrifuge tube and 0.3 ml of 70% methanol internal standard extract was added. The sample was then centrifuged at 12,000 rpm and 4°C for 10 min, before passing the supernatant through a filter membrane (0.22 μm) and storing in a 2 ml brown vial for LC‐MS/MS analysis.

Semi‐quantitative analysis of metabolites was conducted using a UPLC‐ESI‐MS/MS system with the MWDB database (Metware Biotechnology Co., Ltd.) and public databases for qualitative analysis of metabolites [48, 49, 50]. We also detected bacterial metabolites and reference compounds with a Qtrap 5500 triple quadrupole (AB SCIEX) for absolute quantitation and standard curve created at the State Key Laboratory of Crop Stress Biology for Arid Areas (Northwest A&F University).

### Metabolite screening

It has been reported that the α/β hydrolases KAI2 and D14 homology proteins are potential targets of SLs to trigger the pathways controlling the seed germination. Moreover, KAI2 evolved fast and contains seven members in *O. cumana*. To investigate the potential molecular interactions between the top 50 metabolites in the HX79 fermentation broth and *O. cumana*, *Oc*KAI2d2 was selected as the representative binding target to screen the possible active metabolites by Autodock (version 4.2.6) molecular docking [51, 52]. The structure of *Oc*KAI2d2 was obtained by homology modeling based on the template structure of ShHTL7 (pdb code: 5Z82) with 63% sequence identity, which indicates that the quality of modeled structure is reliable. Considering the catalytic reaction mechanism of SLs, the metabolites that can mimic the reaction of SLs are expected to be the potential active molecules. Therefore, the metabolites with a binding mode that could possibly react via the carboxyl C atom on the metabolite and the nucleophilic O atom on Ser 91 were screened out for germination assays.

### Statistical analysis

The α‐diversity index, distance matrix, and PCoA results were obtained using the vegan [53] and ggplot [54] packages in R (version 4.0.0, https://www.r-project.org/). CPCoA plots were obtained using the ImageGP web server (http://www.ehbio.com/ImageGP/). STAMP analysis was conducted to explore the different species among groups using STAMP software. Network analysis was performed with the psych [55] package in R. Co‐occurrence networks were visualized with Gephi software [56]. A Circos plot was produced using circlize [57, 58] and TBtools [59]. The multiple regression model was visualized using the linkET package (https://github.com/Hy4m/linkET) according to the methods described by Jiao [60]. Descriptive statistics were obtained using the agricolae package [61] and GraphPad Prism statistics software [62].

## AUTHOR CONTRIBUTIONS

Yong‐Xin Liu, Yongqing Ma, Quanhong Xue, Jie Gu, James M. Tiedje, Jinming Gao, Zanbo Ding, Jiaxi Liu, Tengqi Xu, Siqi Han, and Jiao Xi did the experiments, analyzed data, and wrote the manuscript. Beilei Lei, Yong‐Xin Liu, Xun Qian, and Lijun Hou analyzed data and revised the manuscript. Xun Qian, Beilei Lei, and Jiao Xi supervised this project. All authors have read the final manuscript and approved it for publication.

## CONFLICT OF INTEREST

The authors declare no conflict of interest.

## Supporting information

Supporting information.

Supporting information.

## Data Availability

All the sequencing data have been deposited in NCBI under submission number SUB11367132 (metagenome sequencing), BioProject accession number PRJNA828360. The data and scripts used are saved in GitHub https://github.com/Xijiao000/iMeta. Supporting Information Materials (figures, tables, scripts, graphical abstract, slides, videos, Chinese translated version and update materials) may be found in the online DOI or iMeta Science http://www.imeta.science/.
