# Supplementary material for: Microbial community roles and chemical mechanisms in the parasitic development of Orobanche cumana
Source: Imeta. 2022 Jun 13;1(3):e31. doi: 10.1002/imt2.31 (PMC10989955; doi:10.1002/imt2.31)
Supplement: Supplementary file 1 — Supporting information. [file IMT2-1-e31-s001.docx]

Supplementary information

**Microbial community roles and chemical mechanisms in the parasitic development of *Orobanche cumana***

**Running title**: *Orobanche cumana* parasitism is influenced by microorganisms

Jiao Xi^1#^, Beilei Lei^1, 2#^, Yong-Xin Liu^3^, Zanbo Ding^1^, Jiaxi Liu^1^, Tengqi Xu^1^, Lijun Hou^4^, Siqi Han^1^, Xun Qian^5^, Yongqing Ma^6^, Quanhong Xue^7^, Jinming Gao^8^, Jie Gu^5^*, James M. Tiedje^5,9^* and Yanbing Lin^1^*

^1^College of Life Sciences, Northwest A&F University, Yangling, 712100, China

^2^State Key Laboratory of Crop Stress Biology for Arid Areas, Center of Bioinformatics, Northwest A&F University, Yangling, 712100, China

^3^Institute of Genetics and Developmental Biology, Chinese Academy of Sciences, Beijing, 100101, China

^4^Department of Natural Resource Sciences, McGill University, Quebec, H9X3V9, Canada

^5^Interdisciplinary Research Center for Soil Microbial Ecology and Land Sustainable Productivity in Dry Areas, Northwest A&F University, Yangling, 712100, China

^6^State Key Laboratory of Soil Erosion and Dry Land Farming, Institute of Soil and Water Conservation, Chinese Academy of Sciences and Ministry of Water Resources, Yangling, 712100, China

^7^College of Natural Resources and Environment, Northwest A&F University, Yangling, Shaanxi, 712100, China

^8^Shaanxi Key Laboratory of Natural Products & Chemical Biology, Northwest A&F University, Yangling, 712100, China

^9^Center for Microbial Ecology, Michigan State University, East Lansing, MI 48824, USA

^#^These authors contributed equally: Jiao Xi, Beilei Lei

*Correspondence: linyb2004@nwsuaf.edu.cn (Yanbing Lin), tiedjej@msu.edu (James M. Tiedje), [gujie205@sina.com](mailto:gujie205@sina.com) (Jie Gu)


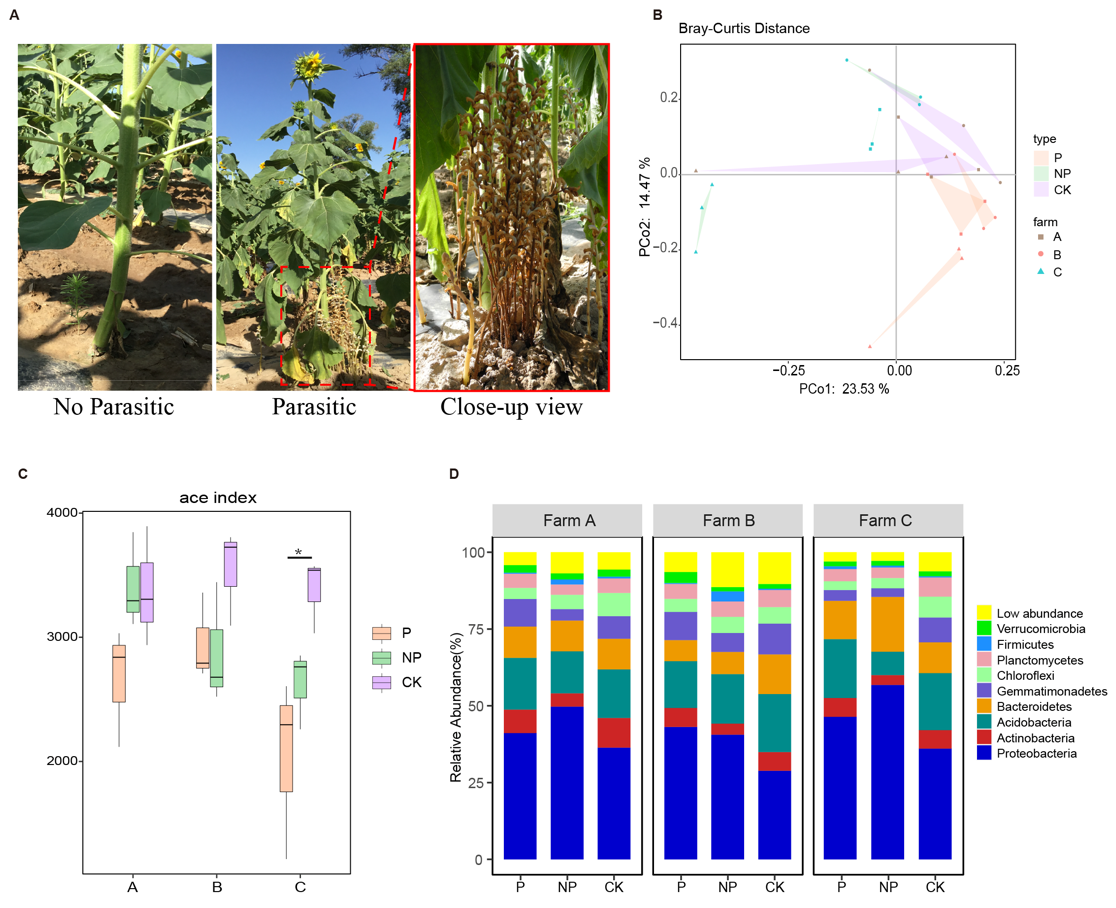


**Fig. S1** Changes in rhizosphere microbiota with *O. cumana* parasitism*.* (A) Sunflower roots without and with *O. cumana* parasites. Approximately 50 *O. cumana* were present on the parasitized sunflower roots. (B) Principal coordinate analysis (PCoA) of the microbial community structures. (C) ACE index for root microbiota. (D) Microbial community compositions at the phylum level. NP: no parasitism, P: parasitism, and CK: corresponding bulk soil.


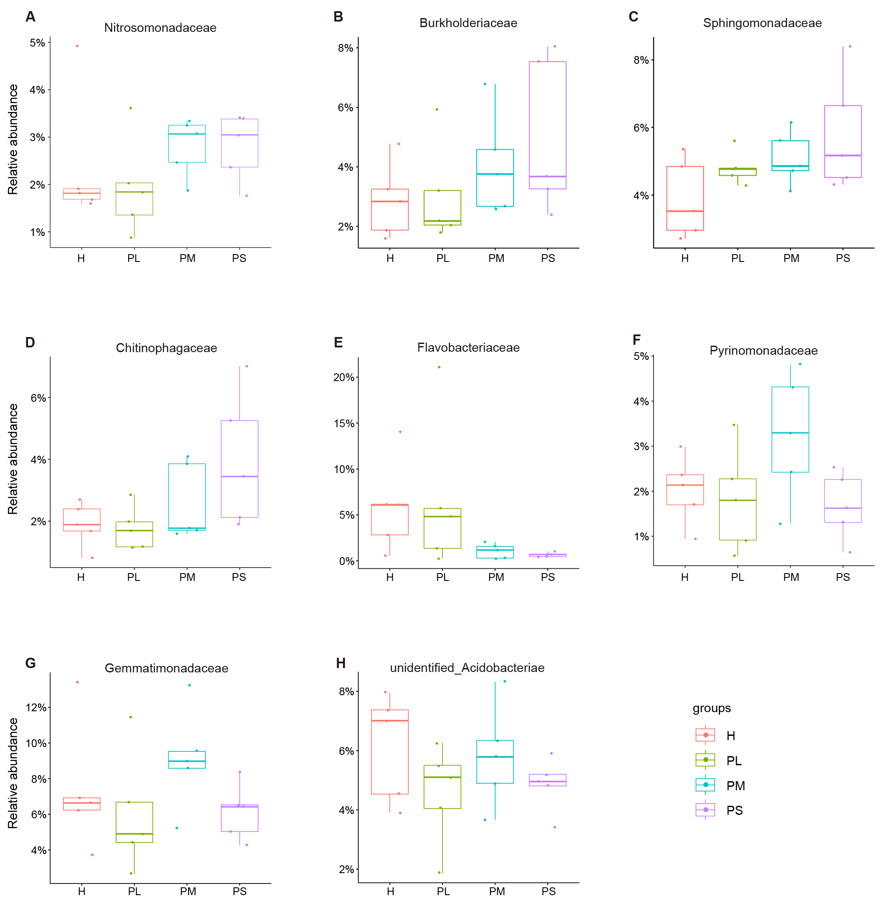


**Fig. S2** Differences in microbial community with *O. cumana* parasitism. Abundances of eight bacterial families in healthy (H), light (PL), moderate (PM), and severely (PS) infected soil samples. The horizontal bars within boxes represent medians. The tops and bottoms of the boxes represent the 75th and 25th percentiles, respectively. The upper and lower whiskers extend to data no more than 1.5 times the interquartile range from the upper edge and lower edge of the box, respectively (n = 5, Wilcoxon test).


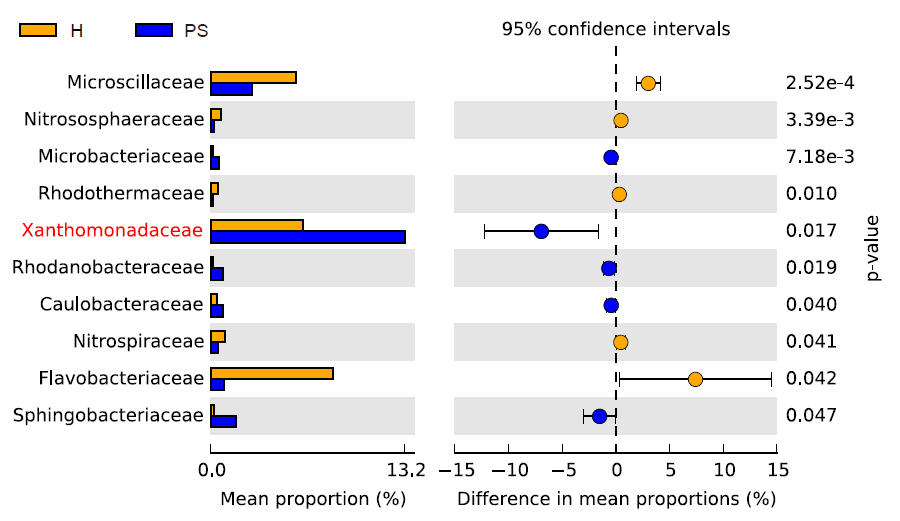


**Fig. S3** Differences in abundances of microbial taxa between H and PS. *P*-values were calculated using two-sided *t*-tests.


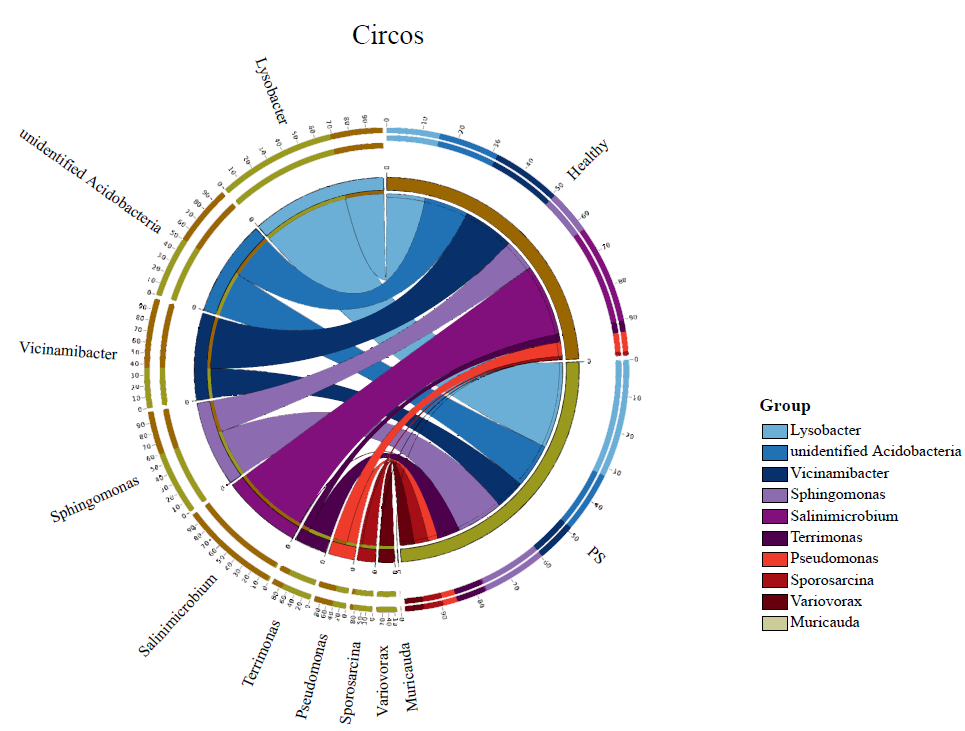
**Fig. S4** Circos plot based on top genera in H and PS soils.


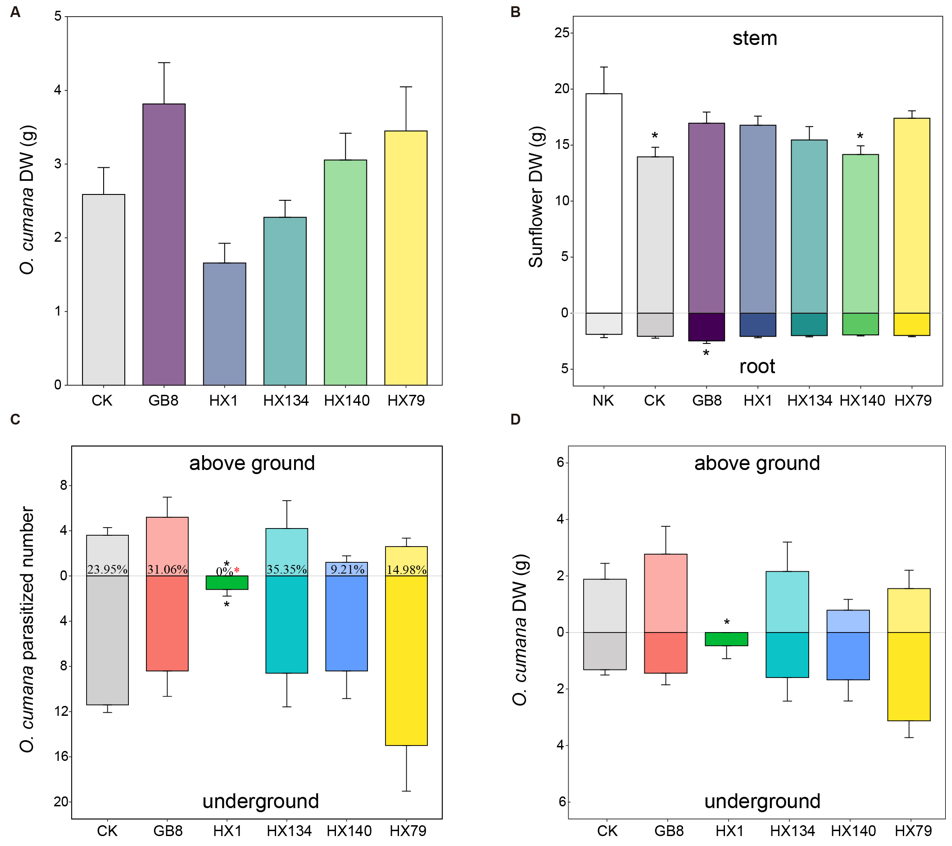


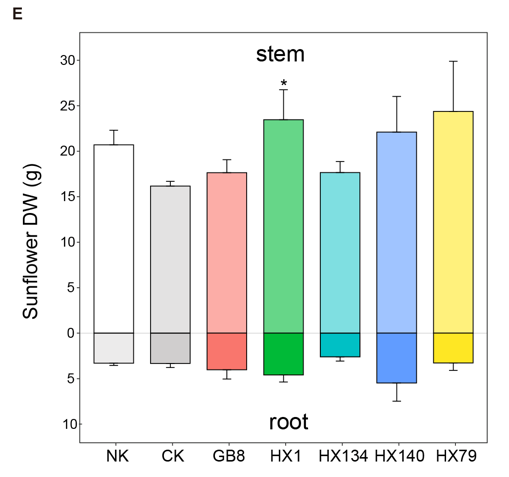


**Fig. S5** Results of pot experiments. Results for first (A–B, n = 13) and second (C–E, n = 5) pot experiments. Under different bacterial treatments, *O. cumana* dry weight (DW) (A, D), sunflower DW (B, E), and parasitized *O. cumana* numbers (C). Percent represents the percentage of aboveground number (%), i.e., the ratio of the aboveground number relative to the number of all parasitic *O. cumana*. CK: *O. cumana* seeds but no bacteria added; NK: no seeds and no bacteria; GB8, HX1, HX134, HX140, and HX79: seeds and corresponding bacteria fermentation liquid added. Different letters denote differences between groups, Kruskal–Wallis test compared with CK, **P* < 0.05.


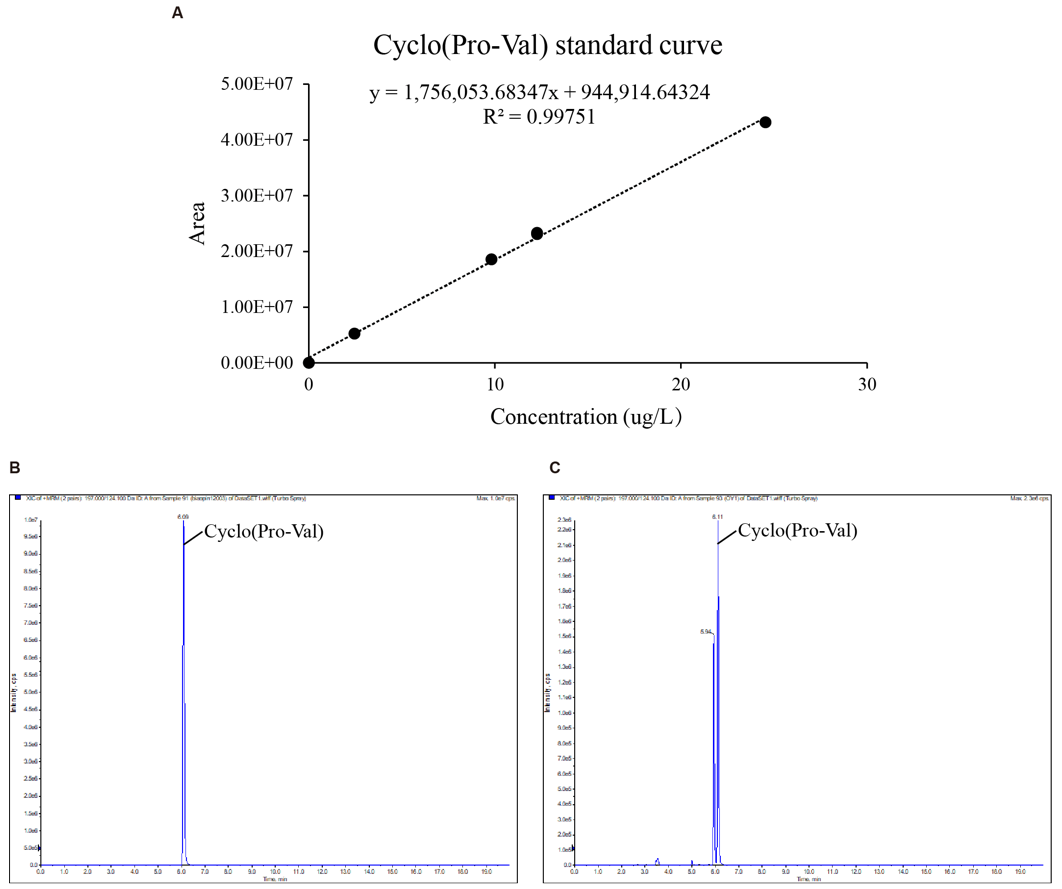


**Fig. S6** Standard curve and LC-MS results. (A) Standard curve calculated and plotted through ordinate (peak) and abscissa (Cyclo(Pro-Val) concentration). LC-MS results for LB medium without HX79 added (B) and HX79 fermented culture solution (C).
